# New Approaches to the Treatment of Chronic Hepatitis B

**DOI:** 10.3390/jcm9103187

**Published:** 2020-10-01

**Authors:** Alexandra Alexopoulou, Larisa Vasilieva, Peter Karayiannis

**Affiliations:** 1 Department of Medicine, Medical School, National & Kapodistrian University of Athens, Hippokration General Hospital, 11527 Athens, Greece; larisatheo@yahoo.gr; 2 Department of Basic and Clinical Sciences, Medical School, University of Nicosia, Engomi, CY-1700 Nicosia, Cyprus; karayiannis.p@unic.ac.cy

**Keywords:** hepatitis B virus, chronic hepatitis B, direct acting antivirals, siRNA, capsid inhibitors, cccDNA modifiers, DNA vaccines, immunotherapy

## Abstract

The currently recommended treatment for chronic hepatitis B virus (HBV) infection achieves only viral suppression whilst on therapy, but rarely hepatitis B surface antigen (HBsAg) loss. The ultimate therapeutic endpoint is the combination of HBsAg loss, inhibition of new hepatocyte infection, elimination of the covalently closed circular DNA (cccDNA) pool, and restoration of immune function in order to achieve virus control. This review concentrates on new antiviral drugs that target different stages of the HBV life cycle (direct acting antivirals) and others that enhance both innate and adaptive immunity against HBV (immunotherapy). Drugs that block HBV hepatocyte entry, compounds that silence or deplete the cccDNA pool, others that affect core assembly, agents that degrade RNase-H, interfering RNA molecules, and nucleic acid polymers are likely interventions in the viral life cycle. In the immunotherapy category, molecules that activate the innate immune response such as Toll-like-receptors, Retinoic acid Inducible Gene-1 (RIG-1) and stimulator of interferon genes (STING) agonists or checkpoint inhibitors, and modulation of the adaptive immunity by therapeutic vaccines, vector-based vaccines, or adoptive transfer of genetically-engineered T cells aim towards the restoration of T cell function. Future therapeutic trends would likely be a combination of one or more of the aforementioned drugs that target the viral life cycle and at least one immunomodulator.

## 1. Introduction

Hepatitis B virus (HBV) is a major public health threat worldwide as nearly 300 million individuals have chronic HBV infection. These patients are at lifelong risk of developing liver cirrhosis and hepatocellular carcinoma (HCC). Currently, two classes of therapeutic agents are approved for the treatment of chronic HBV infection, which can interrupt or prevent this undesirable progression, i.e., the nucleos(t)ide analogues (NAs) and pegylated interferon alpha (Peg-IFN-α). However, both have limitations. On the one hand, the nucleos(t)ide analogues are oral, second generation, reverse transcriptase inhibitors including tenofovir disoproxil fumarate (TDF), entecavir, and the recently approved tenofovir alafenamide (TAF). They effectively suppress HBV DNA levels and have been demonstrated to prevent disease progression to cirrhosis, reverse liver fibrosis, and even cirrhosis, and to reduce but not eliminate, the risk for HCC [1]. However, NAs have little effect on the covalently closed circular DNA (cccDNA), the stable episomal form of the HBV genome which has a very long half-life and can persist for decades in hepatocytes despite effective viral suppression [2]. Hence, NAs do not lead to HBsAg loss, but only to the suppression of viral replication requiring prolonged treatment (indefinite) with concomitant costs [3]. On the other hand, Peg-IFN-α treatment is for a limited period and acts on different phases of the HBV life circle. However, it has a low response rate and the drug is difficult to tolerate [1].

The optimal goal of chronic HBV infection treatment is to attain a “functional cure” of the disease defined by loss of hepatitis B surface antigen (HBsAg) preferably with development of anti-HBs, which results in undetectable HBV DNA in the serum, normalization of liver enzymes, and improved liver histology after treatment cessation [2]. However, in chronically HBV-infected patients, the frequency with which NAs induce HBsAg loss is negligible, particularly in HBeAg-negative patients. Discontinuation of NAs, after a long period of persistent viral suppression, has recently been suggested by the European Association for the Study of the Liver (EASL) [1]. However, according to recent investigations [4], the monitoring of early treatment-free follow-up was demanding and almost half of these patients were not compliant with the requirements. Safety issues were raised in the early treatment-free period due to the possibility of developing acute-on chronic HBV infection, thus one must ensure that the hepatocyte reservoir is at adequate levels. In addition, only a minority of those who stopped NAs, achieved HBsAg loss [4].

It is critical, therefore, to develop new antiviral treatments capable of achieving a functional cure of the disease, and thus reducing the risk of HBV-induced HCC. Ideally, such treatments need to be administered for a finite period of time and at a reasonable cost. Major advances have been made towards understanding multiple steps of the viral life cycle and the mechanisms involved in the evasion of host immune responses that allow the establishment of persistent infection.

In this review, we evaluate preclinical approaches and early clinical investigations regarding potentially new antiviral compounds and innovative treatment strategies in order to enhance both innate and adaptive immunity against HBV which are absolutely necessary if a functional cure is to be achieved.

## 2. Methods

The Medline database, the website www.clinicaltrials.gov, and selected abstracts presented at the EASL and the American Association for the Study of the Liver Diseases (AASLD) meetings regarding new compounds for chronic hepatitis B have been searched, relevant papers reviewed, and summarized.

## 3. HBV Structure and Genomic Organization

Three types of viral particles are present in the serum of an infected individual visible by electron microscopy, i.e., the complete infectious virion or Dane particle and two types of subviral particles known as spheres and filaments [5]. The Dane particle is a spherical particle measuring 42 nm in diameter and consists of an outer envelope made of HBsAg in a lipid bilayer [6]. This encloses the nucleocapsid core of the virus, which in turn contains a single copy of the viral genome covalently linked to the terminal protein of the virus [7]. There is an abundance of subviral particles, which outnumber infectious virions by 100- to 10,000-fold and are exclusively composed of HBs proteins and host derived lipids, lacking any nucleic acid containing cores [8].

The 3.2 kilobases (kb) in length circular partially double-stranded HBV DNA genome contains the four open reading frames (ORFs) of the virus which are the surface (PreS/S), core (C), polymerase (P) and X. These encode a total of seven proteins translated from six co-terminal, unspliced and capped mRNAs ending at a common polyadenylation signal, which is situated in the core ORF. Regulatory elements such as the two enhancers (Enh1 and Enh2), the four promoters (core, S1, S2, and X), the polyadenylation, encapsidation (epsilon), and replication (DR1, DR2) signals are situated within these ORFs and direct the synthesis of the mRNA transcripts through the recruitment of transcription factors which are particularly enriched in hepatocytes [5].

Transcription of the cccDNA occurs in the hepatocyte nucleus [8]. The core promoter is responsible for the synthesis of two longer than genome length mRNAs (3.5 kb), which differ with respect to the start of their 5′ end. The precore mRNA is the longer of the two by a small number of ribonucleotides and contains the initiation codon for synthesis of the precore protein. This is the precursor for provision of the hepatitis B e antigen (HBeAg) following proteolytic processing. The HBeAg is thought to have an immunoregulatory role that facilitates chronic infection establishment and is an important marker of active viral replication [5]. The other transcript is bicistronic and encodes for the core protein (21kD) or hepatitis B core antigen (HBcAg) and the viral polymerase (90kD). It is known as the pregenomic RNA (pgRNA). The core dimerises spontaneously and can form nucleocapsids by self-assembly consisting of 240 copies (120 dimers) of the protein [9]. The polymerase is a multifunctional protein which fulfils a number of roles such as in the facilitation of DNA synthesis during the replication process, reverse transcription, and degradation of the pgRNA. Synthesis of these two proteins is regulated in such a way as to favor the generation of the core molecules required for nucleocapsid formation per single molecule of polymerase packaged with the pgRNA [5].

The S ORF contains three in frame start (ATG) codons that divide the gene into three sections, pre-S1, pre-S2, and S. Thus, polypeptides of three different sizes known as large (L-HBsAg)(pre-S1 + pre-S2 + S), middle (M-HBsAg)(pre-S2 + S), and small (S-HBsAg) are produced [10]. Two transcripts of 2.4 and 2.1 kb are involved in their production, the synthesis of which is under the control of two respective promoters, namely S1 and S2. L-HBsAg is translated from the 2.4 kb transcript, while the M- and S-HBsAgs are translated from the 2.1 kb transcript, the latter through leaky ribosome scanning.

The fourth and smallest ORF encodes for the 17kD HBx protein which is translated from the shortest 0.7 kb in length transcript. This protein is necessary for viral replication and has been implicated in several cellular functions such as cell cycle regulation, signal transduction, transcriptional activation, and DNA repair [5].

## 4. The Life Cycle of the Virus

**Figure 1:**
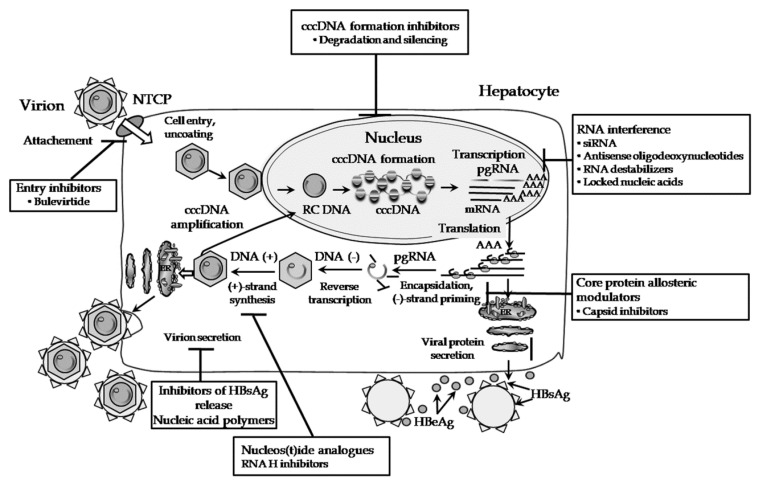
The steps of the hepatitis B virus (HBV) life cycle, from viral entry into hepatocytes to release of mature virions into the extracellular space. The target sites of investigational antiviral agents are noted. NTCP, sodium taurocholate cotransporting polypeptide; cccDNA, covalently closed circular DNA; HBsAg, hepatitis B surface antigen; HBeAg, hepatitis B e antigen.

The HBV life cycle begins following virion attachment to its receptor on the hepatocyte surface, now identified as the sodium taurocholate cotransporting polypeptide (NTCP) [11], which is a bile salt transporter (Figure 1). A stretch of amino acids (2–75) in the pre-S1 domain is involved in virus binding [11]. The interaction between the virus and its receptor can be prevented by neutralizing antibodies against HBsAg forming the basis for the prophylactic vaccination against HBV. Investigation of agents capable of blocking HBV entry and infection of new hepatocytes is currently in progress (see Section 5.1).

Following attachment, two possible pathways have been suggested for cell entry (Figure 1), i.e., endocytosis or fusion of the HBV envelope with the plasma membrane. Either way, the end result is the release of naked nucleocapsids into the cytoplasm and their delivery to the nuclear pores. HBV is thought to use the complex network of the endocytic pathway to reach the nucleus [12]. The nucleocapsid disassembly occurs at the nuclear pore followed by translocation to the nucleoplasm of the released relaxed circular HBV DNA (rcDNA). Within the hepatocyte nucleus, the rcDNA is converted into cccDNA mentioned previously [13]. This involves a number of stages whereby the viral polymerase covalently attached to the 5′ end of the negative-strand (-)-DNA and the short RNA oligomer from the 5′ end of the plus-strand (+)-DNA which is used to prime (+)-DNA synthesis are removed, the variable positive strand is completed, and finally, the ends of the two now complete strands are ligated together. In this form, cccDNA is quite stable and is organized as a minichromosome through association with histones and non-histone proteins, having a plasmid-like structure [14]. Its function is regulated by the activity of various nuclear transcription factors comprising transcriptional repressors, coactivators, and chromatin modifying enzymes [15]. Nearly all elements regulating viral transcription contained within the viral genome and in fact its ORFs, have binding sites for liver specific transcription factors [16]. Hence, the cccDNA utilizes the cellular transcriptional machinery for protein production and viral morphogenesis. The HBV cccDNA molecule is characterized by a high degree of stability, and it can remain in the nucleus for the lifetime of the hepatocyte. Although intrahepatic cccDNA levels can be suppressed during antiviral therapy, this form of HBV DNA appears to be resistant to eradication [17], unless cell death is achieved. Identification of factors affecting the stability and transcriptional activity of the cccDNA might help in the design of new therapeutic approaches aiming at silencing, depleting, or better still eliminating the cccDNA reservoir.

The next step in HBV replication is the packaging of the pgRNA plus the reverse transcriptase into the spontaneously forming nucleocapsids, a process that occurs within the cytoplasm. The encapsidated pgRNA constitutes the template for reverse transcription, mediated by the HBV DNA polymerase and subsequent synthesis of viral rcDNA. The polymerase initiates synthesis of a three nucleotide long DNA primer which is covalently attached to the terminal protein by utilizing the nucleotide sequence of the side bulge of the epsilon encapsidation signal, situated at the 5′ end of the pgRNA [5]. The polymerase-primer complex translocates and hybridizes with the DR1 region at the 3′ end of the pgRNA (terminal redundancy), thereby initiating cDNA synthesis. As the complex proceeds towards the 5′ end of the pgRNA, the pgRNA template is concurrently degraded by the RNase H activity of the polymerase, leaving the nascent newly synthesized (-)-DNA strand of the virus. The RNase H spares from destruction the final 18 or so ribonucleotides which form the primer for (+)-DNA strand synthesis which is initiated once a second translocation event takes place. The ribonucleotide primer hybridizes with the DR1 region at the 5′ end of the newly synthesized (-)-DNA strand [18]. All steps during this stage of the viral life cycle which lead to the synthesis of HBV DNA are targeted by current licensed antiviral treatments using nucleos(t)ide analogues.

Assembly of virus particles occurs in close association with the endoplasmic reticulum (ER) membrane. Mature nucleocapsids bud through the ER membrane into the lumen acquiring in the process their outer envelope containing the HBsAg proteins, already localized there. Ultimately virions are trafficked through the trans-Golgi to the hepatocyte surface via vesicular transport, from where they are released into the extracellular space [19].

## 5. Direct Acting Antivirals

Novel NAs are under development aiming to improve on efficacy from existing ones. For example, besifovir [20] and prodrugs of tenofovir comprising tenofovir exalidex [21], tenofovir disoproxil orotate [22], and metacavir [23] are currently under investigation. However, it is clear that the optimal strategy should aim at targeting multiple steps in the HBV life cycle, so that HBV replication is suppressed and formation of new cccDNA is inhibited. To date, several steps of the HBV life cycle have been considered to be possible targets and have been utilized, for example, inhibitors of the HBV polymerase, blockers of the entry receptor or capsid assembly, and molecules preventing cccDNA formation or inhibiting HBV transcription. Some of these are currently in the clinical or preclinical stage of drug development and are discussed below.

### 5.1. HBV Attachment/Entry Inhibitors

Bulevirtide (Myrcludex B) is a synthetic lipopeptide (myristoylated pre-S1 peptide) that inhibits the entry of HBV into hepatocytes by blocking its binding to the NTCP receptor [24] (Figure 1, Table 1). The drug has been used with or without Peg-IFN-α. In one such Phase 2b clinical trial (MYR203) [25], 60 patients with chronic HBV/HDV (hepatitis D virus) co-infection were randomized 1:1:1:1 into the following four treatment groups: Peg-IFN-α once weekly (*n* = 15)(Arm A), bulevirtide 2 mg once daily (qd) by subcutaneous (sc) injection + Peg-IFN-α qw (*n* = 15) (Arm B), bulevirtide 5 mg qd + Peg-IFN-α (*n* = 15) (Arm C), or bulevirtide 2 mg qd (*n* = 15) (Arm D). All treatments were administered for 48 weeks. A decline of more than 1 log_10_ in HBsAg levels was achieved in 6 and 2 patients from Arms B and C, respectively. Remarkably, 4/15 patients (27%) treated with 2 mg bulevirtide + Peg-IFN-α had undetectable HBsAg levels and 3/4 patients experienced HBsAg seroconversion. A new trial in chronic hepatitis B mono-infection is expected to be completed soon (NCT02888106) (Table 1).

### 5.2. Gene Editing Strategies: cccDNA Formation Inhibitors

Silencing or depleting the cccDNA pool in infected hepatocytes is the goal in new approaches to treatment. Targeted mutagenesis has attracted considerable interest in recent years. This was achieved through the use of sequence-specific RNA-guided nucleases (RGNs) and proteins as a means to cure HBV infection by permanently disabling cccDNA [26,27]. The RGN family includes zinc finger nucleases (ZFNs) [28], transcription activator-like effector nucleases (TALENs) [29,30], and clustered regularly interspaced short palindromic repeats (CRISPR) with CRISPR-associated (Cas) systems [31,32], all showing antiviral efficacy.

The cccDNA is a stable non-integrated minichromosome wrapped in transcriptionally active and inactive chromatin [33]. Designer nucleases can cleave at pretargeted sequences in the HBV genome to result in predefined mutagenesis. Although mutated cccDNA can be transcribed, the resulting mutated viral proteins is not able to engage in viral replication. Hence, HBV cccDNA is an optimal target for nuclease gene editing, due to its episomal minichromosome configuration and sequence stability. More specifically, the efficacy of CRISPR/Cas9 for cleavage and inactivation of the cccDNA, as well as inhibition of hepatocarcinogenesis, has been reported [34,35].

Epigenetic modification, as described above, renders actively transcribed DNA to a transcriptionally inactive status without changing the nucleotide sequence. DNA-binding domains guide epigenetic effectors to predefined sequences of the cccDNA for targeted modifications to occur. Histone modification and cccDNA methylation may induce epigenetic alterations by affecting directly the cccDNA or corresponding histone proteins. Histone acetylation or deacetylation, histone methylation or demethylation, cccDNA methylation, and cccDNA minichromosome acetylation [36] constitute potential epigenetic modifications. Potential HBV DNA modifiers include histone acetyltransferases/deacetylases (HATs/HDACs) [37], lysine methyltransferases [38], protein arginine methyltransferases [39], and DNA methyltransferases (DNMTs) [40], acting in cooperation with viral factors such as HBx and HBcAg (Figure 1). In particular, HBx has been long known to be essential for cccDNA transcription and viral replication through degradation of Smc5/6 which acts as a restriction factor. In this respect, HBx constitutes a reasonable target, interference with which may prevent additional interactions between HBx and virus-related cellular interactions [41].

A novel first in class molecule cccDNA destabilizer, ccc_R08, targeting preexisting viral genome reservoirs was presented in [42]. This small molecule showed a robust and sustained suppression of HBsAg, HBeAg, HBV DNA, and HBV RNA levels in serum, and reduction of cccDNA levels in the liver of an experimental mouse model transduced with circular DNA able to replicate HBV using a cccDNA-dependent mechanism similar to that observed in humans.

### 5.3. RNA Interference (RNAi)

RNA interference is another potential approach to treatment, as all five viral mRNA transcripts could be targeted by a single compound with a knock-on effect on synthesis of all viral antigens. Small interfering RNA molecules (siRNAs) (Figure 1, Table 1) are a class of double-stranded RNA molecules, 20–25 base pairs in length, whose function is to regulate gene expression [43,44]. Interaction with the Ago2/RISC complex degrades one strand, whilst the remaining one exerts its function by binding to the complementary mRNA target leading to its degradation via an RNA-induced silencing complex (RISC)-mediated process [45]. Such compounds could theoretically affect the HBV DNA level and also levels of HBsAg, HBeAg, and hepatitis B core-related antigen (HBcrAg), thus, causing a reversal in immune exhaustion (see later), allowing effective HBV-specific immune responses to be raised, and finally leading to HBsAg seroconversion and functional cure. The delivery of siRNAs at their site of action requires the use of appropriate carriers rendering them amenable to intravenous administration. Different methods to deliver HBV-specific siRNA to infected liver cells are now used in clinical studies.

GalNAc-siRNA is delivered by the conjugated targeting ligand GalNAc that enables subcutaneous delivery. The ligand interacts with the asialo-glycoprotein receptor which is highly expressed on hepatocytes. The first data on this novel siRNA approach were reported during the EASL ILC 2018 [46]. Agent AB-729 has shown promising results in a Phase 1a/1b clinical trial, as it targets all four ORFs of HBV. It is effective against HBV genotypes A-H and selected NA-resistant variants and is not cytotoxic up to the highest dose tested. Data from preclinical studies support monthly dosing which leads to a significant drop in serum HBsAg, HBeAg, and HBV DNA levels. This agent will next move to clinical safety studies. ARB-1467 (NCT02631096) is a lipid nanoparticle (LNP) compound containing three siRNAs which target three distinct sites for post-transcriptional HBV gene suppression, including HBsAg. HBsAg reductions were achieved following single and multiple dose administration [47]. More results concerning this compound are eagerly awaited.

The data from another compound, the JNJ-3989 (formerly ARO-HBV), was recently presented. The drug was able to silence HBV RNA from cccDNA and host-integrated viral DNA [48]. In a phase 2 clinical trial, in 40 patients, the drug was demonstrated to reduce all measurable viral products, including HBsAg in HBeAg-positive or -negative patients. More specifically, NJ-3989 rapidly reduced HBsAg to levels possibly associated with improved chances of HBsAg seroclearance (<100 IU/mL) in 88% of patients and <10 IU/mL in 43% of patients. More recently, at the EASL Meeting, 2020, the results confirmed previous findings that HBsAg levels declined equally with doses from 100 to 400 mg given once every four weeks and for three doses combined with a NA; 98% of the patients achieved a ≥1.0 log_10_ reduction in HBsAg [49].

Antisense oligonucleotides, RNA destabilizers, and locked nucleic acids are under investigation [50], (Figure 1, Table 1). In a phase 2a, multicenter, randomized study in 66 HBeAg-positive and -negative NA suppressed patients, GSK3389404 (antisense oligonucleotide) caused a rapid drop in HBsAg levels (0.7 log_10_) after three months of the maximum dose of 120 mg weekly [51].

The major concern was the delivery platform, which raised potential safety issues. Thus, clinical trials using the ARC-520, ARC-521, AB-506, and ARB-1740 compounds were terminated.

### 5.4. Ribonuclease H (RNase H) Targeting

PgRNA which is encapsidated in core particles is the template for (-)-strand DNA synthesis by reverse transcription. In the process, the pgRNA template is degraded by the RNase H domain of the polymerase. Inhibitors of the RNase activity will prevent this from happening and in addition will avert subsequent synthesis of the (+)-strand DNA as described before. Recently, a number of chemical classes of potential RNase H inhibitors have been identified including a-hydroxytropolones, N-hydroxyisoquinolinediones, and N–hydroxylpyridinediones [52,53] (Figure 1, Table 1). This opens up the possibility of their use, either alone, or more likely in combination with other existing DAAs or new ones that may be developed.

### 5.5. Nucleocapsid Assembly Inhibitors and Core Inhibitors

The HBV core protein plays a central role in the viral replication cycle starting with uncoating and release of rcDNA, delivery to the nuclear pore basket, nucleocapsid formation and packaging of pgRNA, and finally interaction with HBsAg during the end stages of morphogenesis. There is indirect evidence that the protein binds to cellular promoters and regulates gene expression (binds the cccDNA and modifies cccDNA nucleosome spacing) [54,55]. In this respect it constitutes another attractive antiviral target.

The following two classes of core protein allosteric modulators (CpAM) have been discovered: the heteroaryldihydropyrimidines (HAPs) (Type I CpAMs) and the phenylpropenamides (PPAs), sulfamoylbenzamides, and several other chemotypes (Type II CpAMs) (Figure 1, Table 1).

HAP derivatives, misdirect core protein dimers to assemble aberrant non-capsid polymers, leading to the degradation of the core protein [56]. GLS4 [57] is a representative compound of the HAP family.

Type II CpAMs were found to accelerate formation of capsid assembly, possibly at an inappropriate time and place, thereby preventing pgRNA encapsidation and, instead, inducing the assembly of empty capsids [58].

In vitro, GLS4 inhibited virus accumulation in the supernatant of hepatic cell lines. This was tested in vivo in nude mice inoculated with HepAD38 cells, which then grew out as tumors, resulting in viremia. Treatment of mice with GLS4 and BAY 41-4109 caused a strong and sustained drop in HBV DNA to about the same extents both during and after treatment [59].

An oral type I HBV core inhibitor molecule, RO7049389, induces formation of abnormal HBV core protein aggregates which are, then, depleted, leading to disruption of viral assembly and potent inhibition in HBV replication. In vivo, it induced a robust HBV DNA decline of about 3.0 log_10_ copies/mL over 56 days of dosing. An ongoing Phase 1 study is investigating the safety, tolerability, pharmacokinetics, and anti-HBV activity of this compound [60]. The food effect on dosing was explored in single and multiple ascending doses (SAD vs. MAD). The anti-HBV effects of RO7049389 were recorded in untreated chronic HBV patients (ALT< 5 × ULN, without liver cirrhosis) divided into five groups according to dose. After four weeks of drug administration, a decline in HBV DNA and HBV RNA was observed across all five cohorts; 81.3% of patients attained HBV DNA levels lower than the lower limit of quantitation. However, no HBsAg change was observed during four weeks of treatment.

Interestingly, studies in multiple in vitro and in vivo HBV experimental models have found that the type I core inhibitor HAP_R01 reduced HBV DNA and HBeAg levels through causing misassembly of its 22 kDa precore protein precursor [61]. It seems that HAP_R01, as well as other similar CpAMs, have the potential to achieve higher anti-HBe seroconversion rates than the currently approved therapies for patients with CHB.

The results from a type II HBV core inhibitor molecule, EDP 514 in a mouse model, were recently reported [62]. The molecule was shown to reduce HBV DNA and HBV RNA and to induce a slight decline in HBsAg and HBeAg levels. EDP 514 had synergistic antiviral effect when combined with ETV, or TFV, or the type I HBV core inhibitor GLS4. Other CpAM compounds under investigation are AB-423, ABI-H0731, JNJ-56136379, and JNJ-6379 (Table 1). A phase 1 trial of ABI-H0731 in combination with entecavir provided safety, as well as efficacy, data which showed that the drug was well tolerated with a decrease in HBV DNA and HBV RNA levels [63]. When used in combination therapy with a NA together with oral JNJ-6379 (CpAM) 250 mg for 12 weeks and three 200 mg subcutaneous doses of JNJ-3989 (RNAi) on days 1, 29, and 57, it resulted in a marked decline in HBsAg levels [64].

### 5.6. HBsAg Release Inhibitors

Nucleic acid polymers (NAPs) (Figure 1, Table 1) use phosphorothioated oligonucleotides to target apolipoprotein interactions involved in the assembly and release of HBV subviral particles (SVPs), which are made up of HBsAg. These work in a sequence independent manner to block SVP formation inside infected hepatocytes and their subsequent secretion [65,66]. As SVPs account for greater than 99.99% of HBsAg in the blood, NAPs constitute an effective means of clearing HBsAg from the serum of patients with chronic HBV infection [67].

HBsAg has important immunosuppressive effects on HBV infection which have been shown to block both adaptive and innate immune mechanisms (see later). Elimination of HBsAg from the serum of patients removes this immunosuppressive effect [68], thus, overcoming anergy or T cell exhaustion. Thus, an important additional effect of removal of HBsAg from the serum is to amplify the effect of Peg-IFN-a [69,70].

Some of the compounds comprising REP-2055 [71], REP-2139, and REP-2165 were shown to prevent the release of HBsAg from infected liver cells. In a REP 301 and REP 301-LTF trial [72], twelve HBV/HDV co-infected patients received 500 mg REP 2139-Ca intravenously once per week for 15 weeks, followed by combined therapy with 250 mg intravenous REP 2139 and Peg-IFN-α once per week for another 15 weeks, then monotherapy with Peg-IFN-α for 33 weeks. The treatment with the NAP REP 2139 resulted in rapid and effective clearance of HBsAg and seroconversion to anti-HBs in 42% of patients, which was maintained after treatment discontinuation.

Although REP 2139-Ca has been shown to be safe in humans, it accumulates in the liver with repeated dosing. REP 2165 is a version of REP 2139, which is designed to have lower liver accumulation while maintaining its antiviral activity intact. The antiviral efficacy of REP 2165 has been shown to be comparable to REP 2139 in a preclinical model of HBV infection with significantly less accumulation in the liver. As such, REP 2165 is expected to have comparable antiviral efficacy in humans with reduced liver accumulation during treatment.

In a REP 102 study, twelve HBeAg-positive patients received therapy for a period of 32–58 weeks with REP 2139-Ca. Nine patients with a >2 log_10_ drop in HBsAg and HBV DNA levels from baseline were eligible to receive add-on immunotherapy with thymosin alpha and/or PegIFN-α. Those with reductions in HBV DNA and HBsAg levels of <1 log_10_ following 24–40 weeks of REP 2139-Ca monotherapy were classed as nonresponders and received entecavir. Eight out of nine patients seroconverted to anti-HBs having experienced reduction in levels or loss of HBsAg, indicating functional control of HBV infection. The investigators studied the kinetics of HBsAg quasispecies during REP 2139-Ca treatment and found a decline in HBsAg quasispecies complexity in responders as compared with non-responders [73].

A phase 2 clinical trial (REP 401) (NCT02565719) combining REP 2139-Mg or REP 2165-Mg with conventional dosing of Peg-IFN-a and TDF was presented in London, UK in an HBV endpoint meeting, in 2019 [74]. Twenty-four weeks of lead-in TDF (300 mg PO qD) therapy was followed by randomization (1:1) into experimental and control groups (20 patients each). The experimental group received 48 weeks of triple combination of TDF, peg-IFN-a, and REP 2139-Mg or REP 2165-Mg (1:1, 250 mg IV infusion qW). The control group received 48 weeks of TDF + peg-IFN-a, but all patients had crossed over to 48 weeks of NAP experimental therapy in the absence of a 3 log_10_ decline in HBsAg after 24 weeks of TDF + peg-IFN-α. At the end of treatment with triple combination therapy, 67% achieved an HBsAg titer <1 IU/mL. Functional cure (HBsAg undetectable) was achieved in 41% of 34 patients who completed the 24–48 weeks of follow-up off therapy. Responses to REP 2139-Mg and REP 2165-Mg were indistinguishable [75]. It is worth noting that more than 90% of patients had ALT flares. Flares were more pronounced amongst those who achieved a functional cure. Flares were self-resolving in asymptomatic patients.

## 6. Immunotherapy

Recovery from acute HBV infection acquired in adulthood is spontaneous and relies on a broad and multispecific immune response. The role of the innate immune response in the natural clearance of HBV infection has been less than clear. However, recent developments have suggested that natural-killer (NK) cells had a role in early HBsAg clearance, whilst other innate immune cells may be involved in the regulation of immune action [76]. Non-cytolytic suppression of virus replication may be responsible for a decline in viraemia decline prior to symptom appearance, mediated perhaps by IFN-a likely secreted by NK and NKT cells. Experimental animal studies have indicated that an HBV-specific cytotoxic T cell response (CTL), which was strong and polyclonal in nature against viral proteins such as HBcAg, was essential for clearance of infected hepatocytes [77]. Neutralizing anti-HBs are responsible for clearance of free virions and prevention of their attachment to hepatocytes.

**Figure 2:**
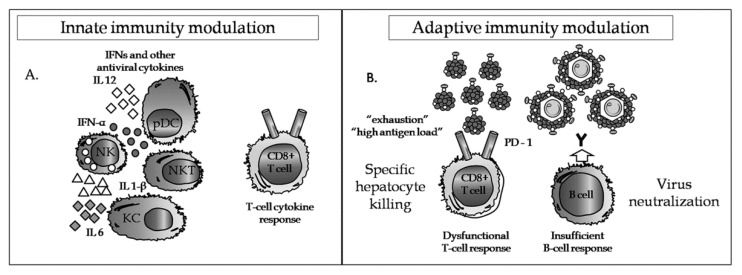
(**A**) HBsAg, HBeAg, and HBx can interfere with the innate immune response and in particular components of signal transduction pathways or other processes which, in turn, can disregulate IFN and antiviral cytokine production by effector cells such as natural killer (NK), NK T cells (NKT), kuppfer cells (KC) and plasmacytoid dendritic cells (pDC). Such events may inhibit CD4+ and CD8+ T cells; Interferons (INFs (**B**) The adaptive immune response relies on the production of virus neutralizing antibodies and cytotoxic T cells for lysis of infected hepatocytes. However, chronic HBV infection is characterized by the loss or functional exhaustion of HBV-specific CD8+ T cells due to high levels of HBs antigenaemia and failure to neutralize circulating virions as a result of an insufficient B-cell response. Programmed cell death protein-1 (PD-1).

The progression to chronic HBV infection appears to be the result of dysfunctional HBV-specific immune responses. The innate immune response at the onset of infection is not robust enough and IFN-α is lacking as NK cells and plasmacytoid DCs (pDCs) are deficient or dysfunctional (Figure 2). Downstream, maturation of antigen presenting cells such as dendritic cells (DCs) is disrupted by high levels of HBsAg or HBeAg, inducing tolerance. Moreover, chronic inflammation in the liver caused by infiltrating monocytes/macrophages, NK, NKT, and T cells (including regulatory T cells) being the result of the presence of proinflammatory cytokines, contributes to immunopathology [78]. Furthermore, CTL responses are narrow and monospecific and inadequate in clearing infected hepatocytes. Intrahepatic CD8+ T cells express programmed cell death protein 1 (PD-1) which suggests that they may become exhausted in the presence of cognate antigen expression (Figure 2).

Bearing all the above in mind, any new therapeutic approaches intending to activate the immune response should aim to promote death of infected hepatocytes, avert hepatocyte infection, establish long-term viral control, and more importantly lead to elimination of the cccDNA pool. Experience has shown that development of anti-HBs, either through spontaneous or antivirally induced seroconversion, did not necessarily lead to the elimination of any residual cccDNA pools in hepatocytes. If such patients receive immunosuppressive therapy to treat inflammatory diseases or lymphomas (steroids, Rituximab, etc.) [78], then, loss of immunological control may lead to HBV reactivation. Thus, any immunotherapeutic approaches directed against HBV must be such so as to precipitate true functional cure, but at the same time be mindful of potential uncontrolled hepatic flares and autoimmune phenomena. In this respect, both arms of the immune response, innate and adaptive, could be targeted in an attempt to overcome the immunological failures described above.

### 6.1. Interventions that Activate the Innate Immune Response

#### 6.1.1. Toll-Like Receptor Agonists

Toll-like receptors (TLRs) constitute the first line of defense against invading microorganisms, in that they sense pathogen-associated molecular patterns (PAMPs, components) leading to the production of cytokines through signal transduction pathways. TLR-7 and TLR-8 agonists are involved in endogenous IFN production, induction of IFN-stimulated genes (ISGs), and activation of other signaling cascades such as the JAK/STAT pathway. The TLR-7 agonist GS-9620, when first tested in the human hepatocyte cell line HepaRG and primary hepatocytes infected with HBV, showed a durable suppression of HBV replication through induction of Type I IFN but no reduction in cccDNA levels [79]. Similar results were obtained in the woodchuck and chimpanzee animal models [80,81], but when used in patients with HBV who were at the time suppressed with NA treatment, there was no significant effect on HBsAg levels [82,83]. Combination treatment with another TLR-7 agonist (RO7020531) and a capsid assembly modulator RO7049389 produced a significant reduction in HBV DNA and HBsAg levels in a mouse infected with a recombinant adeno-associated virus (AAV-HBV infection mouse model) [84]. In Chinese healthy human volunteers, single and multiple ascending doses resulted in IFN-a-induced cytokine production and induction of ISGs [85]. Its efficacy in combination with other antivirals remains to be determined (Table 2).

#### 6.1.2. Retinoic Acid-Inducible Gene-1 (RIG-I) Agonists

RIG-I constitutes an intracytoplasmic PAMP receptor interacting with viral double stranded RNA derived from RNA viruses. Once activated, it leads to signal transduction through protein kinase complexes and activation of NFκB and IRF3 transcription factors, which in turn migrate to the nucleus where they activate ISGs leading to production of IFN-a and other cytokines that initiate antiviral immunity. Recently, it has been reported that the epsilon-encapsidation signal present in pgRNA was recognized by RIG-I leading to the production of type III IFNs rather than type I. In addition, it has been noticed that RIG-I counteracted the interaction of epsilon with the HBV polymerase causing suppression of HBV replication [86]. Inarigivir (SB 9200), a RIG-I/NOD-2 agonist, was investigated in the ACHIEVE study (Study Evaluating the Safety, Pharmacokinetics, and Antiviral Efficacy of SB 9200 in Subjects Infected with Chronic HBV) which enrolled 80 treatment naïve non-cirrhotic CHB patients. The patients were randomized to receive ascending doses of the drug from 25 to 200 mg or placebo for 12 weeks, followed by a switch to TDF for another 12 weeks. HBV DNA and RNA reductions were achieved in both HBeAg-positive and -negative patients in a dose dependent manner, that being greater in the latter. An HBsAg reduction of >0.5 log_10_ at 12 or 24 weeks was seen in 22% of patients. Further study of Inarigivir at the 400 mg dose with TDF is currently under way [87].

#### 6.1.3. Stimulator of Interferon Genes (STING) Agonists

Stimulator of Interferon Genes (STING) is the main molecule involved in signal transduction following intracellular pathogen DNA recognition such as cyclic GMP-AMP Synthase (cGAS). Using synthetic agonists for activation of the cGAS-STING pathway, it has been shown that IFN production was induced in a mouse model persistently infected with HBV which led to the inhibition of HBV replication [88,89]. Such agonists may offer an additional avenue in progressing with innate immunotherapy (Table 2).

#### 6.1.4. Checkpoint Inhibitors

PD1 is highly expressed on HBV-specific T cells and, as a result, is associated with the dysfunctional T cell responses mentioned above [90,91]. Checkpoint inhibitors directed against PD-1, therefore, may help to restore T cell dysfunction. In a recent 1b trial, the efficacy of the PD-1 inhibitor nivolumab was investigated in 24 NA-suppressed HBeAg-negative chronically infected patients. Patients received either 0.1 or 0.3 ng/kg once, or two doses of the latter in combination with the therapeutic vaccine GS4774 at baseline and 4 weeks later. Patients receiving the higher dose had a significant reduction in HBsAg levels from baseline, whilst one patient receiving the combination lost HBsAg [92]. Further testing of these compounds in the clinical setting would indicate whether they could be taken to the next step (Table 2).

CD8+ T cells of exhausted phenotype in chronic HBV infection present other than PD-1 additional co-inhibitory molecules such as CTLA-4, CD244/2B4, Tim-3, and LAG-3. Of these, blockade of the first two in in vitro studies achieved restoration of immune function and increased proliferation of peripheral blood and intrahepatic CD8+ T cells [93,94]. Moreover, CTLA-4 blockade with ipilimumab suggested a positive effect against HBV in patients with advanced melanoma [95]. CTLA-4 blockade appears to act on other immune cells such as follicular helper T cells whose activity was enhanced in an HBV mouse model [96].

### 6.2. Modulation of the Adaptive Immune System

#### 6.2.1. Therapeutic Vaccination

Previous attempts at different times during the past two decades, using existing prophylactic vaccines with varying vaccination strategies, have failed to restore HBV-specific immunity in chronically infected patients. When used in animal models, they provided promising results, which were not replicated in human subjects [97,98,99]. Such vaccines which employed an adjuvant, although capable of stimulating a robust B cell response, failed to induce the cytotoxic arm of the immune response which was necessary to achieve a therapeutic outcome. In an attempt to overcome this disadvantage, DNA vaccine strategies were introduced employing coding sequences of HBsAg but, once again, yielded poor results in patients virally suppressed by analogue treatment. Notably, they failed to lead to either HBeAg or HBsAg seroconversion [100,101]. Of similar faith, was the use of DNA prime followed by poxvirus boost, the latter containing the preS/S encoding region. Although promising results were obtained once again in the chimpanzee animal model [102], in a phase IIa trial this approach did not induce sustained T cell responses or achieve a sizeable reduction in viral load in chronic HBV carriers [103].

#### 6.2.2. More Recent Approaches

ABX-203 (HeberNasvac) is a vaccine containing both HBsAg and HBcAg administered intranasally (Table 2). In a phase III study, 160 patients were randomized 1:1 to receive either the vaccine or Peg-IFN-α; 10 doses of the vaccine in two cycles or Peg-IFN-a for 48 weeks. At the end of treatment, HBV DNA suppression was similar between the two groups (59.0% versus 62.5%), whereas 24 weeks later, patients with viral load below 250 copies/mL were more frequent in the vaccine group (57.7%) than the Peg-IFN group (35%). Moreover, HBeAg seroconversion was achieved in 5/14 vaccine recipients (35.7%) as opposed to 3/16 (18.7%) in the Peg-IFN-a group [104]. Similarly, in a phase I trial in Cuba, six CHB patients unresponsive to Peg-IFN treatment, received the vaccine as described and after a five-year follow-up, HBeAg loss was achieved in three HBeAg positive patients, in two cases with seroconversion to anti-HBe. An undetectable viral load was present in 5/6 and seroconversion to anti-HBs in two patients [105].

INO-1800 is a DNA-based HBV vaccine encoding HBsAg and a consensus sequence of HBcAg (Table 2). In a preclinical study, the vaccine induced antibodies to HBs and a robust cell-mediated immunity in both mice and Rhesus macaques. The vaccine-induced responses were broadly distributed across multiple antigenic epitopes [106]. The results of an open-label, dose escalation study evaluating the safety, tolerability, and immunogenicity of the vaccine with or without INO-9112 which encodes human IL-12 to 90 CHB patients virally suppressed with either entecavir or tenofovir are eagerly awaited (Inovio, 2018). Widening the antigenic repertoire, vaccine HB-110 was designed to include constructs containing the S- and L-HBsAgs, the core and polymerase, and the human IL-12 coding sequences. In a phase I clinical study, 27 CHB patients were randomized to receive the vaccine with adefovir or adefovir alone. Although HB-110 was shown to be safe and tolerable in CHB patients, HBV-specific T cell responses appeared to be quite weak [107]. Both INO-1800 and HB-100 need to be administered by in vivo electroporation to maximize vaccine antigen expression and immunogenicity, a method that is not easy to perform and likely to cause problems.

#### 6.2.3. Vector-Based Vaccines

GS-4774 is a recombinant, heat inactivated yeast-based vaccine (*Saccharomyces cerevisiae*) which encodes HBsAg, HBcAg, and HBx. In a phase II study, 178 CHB patients with no cirrhosis and virally suppressed with approved oral antivirals were randomized to continue antiviral therapy alone or in addition receive 2, 10, or 40 yeast units of GS-4774 subcutaneously every 4 weeks, until week 20. Mean HBsAg declines from baseline to week 24 or 48 were similar between the groups. Five HBeAg-positive patients that received GS-4774 experienced HBeAg loss vs. none in the control group. The authors concluded that, although the vaccine was safe and well tolerated, it did not result in a clinical benefit [108]. Similar results were obtained in a separate phase 2 study in 195 patients virally suppressed by tenofovir treatment. Randomization and dosing was as described above. Nevertheless, although vaccination did not reduce levels of HBsAg, there was an increase production of IFN-α, TNF-α, and IL2 by CD8+ T cells exposed to antigenic peptides. This suggests that the strong immune stimulatory effect on CD8+ T cells recorded may be of benefit if the vaccine is used in combination with other antiviral agents in development to boost its antiviral potential [109].

TG-1050 is a non-replicating adenovirus five-based vaccine encoding vector which expresses a fusion product containing the HBV polymerase and domains from core and surface antigens, and which in mice has been shown to be immunogenic and have an antiviral effect (Table 2). A randomized, double blind, placebo-controlled study included two sequential phases, i.e., one single dose cohort (*n* = 12) and one multiple (3) doses cohort (*n* = 36) in virally suppressed patients by analogue therapy. The three doses used were 10^9^, 10^10^, 10^11^ virus particles of TG1050. All doses were well tolerated in both cohorts and the vaccine was capable of inducing IFN-γ producing T cells that targeted one to three encoded antigens, more so with the 10^10^ virus particle dose [110].

The use of viral vectors that carry HBV protein encoding regions are themselves immunogenic leading to the generation of anti-vector antibodies. As such approaches entail multiple dosages, subsequent injections may be less efficient because of the presence of pre-induced antibodies. Approaches whereby booster immunizations involve the use of heterologous vectors may be indicated in an attempt to maximize efficacy such as modified vaccinia Ankara strain or cytomegalovirus.

#### 6.2.4. Adoptive Transfer of Genetically Engineered T Cells

Whether T cell exhaustion can be overcome and to which extent immune function can be restored in order to control HBV remains unknown. One approach in addressing this is the generation in vitro of functionally efficient T cells derived from patients intended for treatment, with specificity against HBV, which can, then, be re-infused in an attempt to overcome immunological anergy.

Evidence that adoptive transfer of engineered antigen-specific T lymphocytes can work has been forthcoming from cases of bone marrow and solid organ transplantation. Bone marrow from subjects who cleared HBV infection spontaneously, transplanted into chronic HBV-infected patients, led to effective control of the virus [111]. Similarly, virus clearance was achieved in a liver transplant patient with resolved HBV infection who received an HBsAg positive graft [112]. Thus, adoptive T cell therapy using autologous T cells genetically engineered to express HLA class I restricted epitopes is a feasible approach to use against HBV. T cells isolated from the peripheral blood of CHB patients can be expanded and activated in vitro, and then engineered to develop specificity against HBV through the use of viral vectors that encode HBV-specific T cell receptors able to recognize HBV protein peptides in association with HLA class I antigens. Re-infusion of such modified cells have been tested in both HBV transgenic mice and patients with HCC due to chronic HBV infection [113,114] has provided encouraging results. Other approaches that have been employed in this respect include transient expression of HBV-specific TCRs through electroporation of appropriate mRNAs [115], and the generation of HBV-specific T cells with restricted lytic capacity but having efficient antiviral properties (e.g., activation of APOBEC3) [116].

An alternative approach entails the use of T cells which can recognize HBV-infected cells independently of patient HLA haplotype. Such cells are engineered to express a chimeric antigen receptor (CAR) that includes an HBV-specific antibody fragment with CD28 (co-stimulatory molecule) and the zeta intracellular domain of CD3. Delivery of these chimeric receptors using retroviral vectors have been shown to recognize and kill HBV-infected hepatocytes expressing HBsAg and eliminate cccDNA, if using CAR T cells against HBsAg [117]. These can localize in the liver in a transgenic mouse model and impact HBV replication [114]. HBV DNA and HBsAg level reduction was achieved in an HBV-infected immunodeficient mouse model with chimeric human livers [118].

Undoubtedly, the above approaches hold promise, but research work is still at an early stage of development. Moreover, their implementation for clinical use presents a number of practical difficulties that include production of large quantities of engineered T cells, a process that is strictly regulated, the cumbersome technological manipulations that are necessary and the need for skillfully trained technologists.

## 7. Conclusions

Despite viral suppression by nucleos(t)ide analogues, there are many barriers to achieve a “cure” of HBV infection. First, HBV persists in the hepatocyte nucleus through continuous replenishment of the cccDNA pool, with a long half-life and the integrated forms of viral DNA. Second the defective immune response, and especially the defective CD8+ responses and the inefficient innate immune response, prevent HBV-infected hepatocytes from been cleared. To switch the balance towards elimination of HBV infection, decreased recycling of the cccDNA reservoir, non-cytolytic and/or cytolytic cccDNA elimination and increased non-infected hepatic cell division are needed. However, even with the hypothetical achievement of cccDNA elimination, integrated viral sequences would still be present in hepatocytes, and thus maintain the risk for hepatocellular carcinoma development.

HBsAg loss with or without anti-HBs seroconversion is for the time-being one of the most desired endpoints of ongoing clinical trials. More advances have been made in the past few years towards understanding the viral life cycle and viral pathogenetic mechanisms leading to the identification of new therapeutic viral targets. Targeting HBsAg, either by inhibiting entry of HBV into hepatic cells or by blocking assembly and release of subviral particles, attempt to decrease serum HBsAg levels, thus, preventing its immunosuppressive effects and driving of T cell exhaustion. New research approaches aim to develop molecules that block formation of viral products (direct acting antivirals), as well as other agents acting on human innate or adaptive immunity and, particularly, those achieving immune restoration (immunomodulators).

The current approach in the treatment of chronic HBV infection is to use a combination of multiple drugs including a backbone of a nucleos(t)ide analogue, one or more new direct acting antiviral drugs, and at least one immunomodulator. The most efficient antiviral strategy and the type of potent antivirals or immune therapeutics used as monotherapy or in combination remain to be established.

## Figures and Tables

**Table 1:** Novel direct acting antivirals in clinical trials in chronic HBV infection.

Drug Family	Drug Name	Trial N	Phase	Status	Sponsor
**Entry inhibitor**	Bulevirtide (Myrcludex B)	NCT02881008	2	Completed	Hepatera Ltd.
		NCT02637999	2	Completed	Hepatera Ltd.
		NCT04166266	2	Not yet recruiting	Inserm-ANRS
		NCT03546621	2	Completed	Hepatera Ltd.
		NCT02888106	2	Recruiting	Hepatera Ltd.
		NCT03852719	3	Recruiting	MYR GmbH
		NCT03852433	2	Recruiting	MYR GmbH
**Small interfering RNA molecules (siRNAs)**	VIR-2218	NCT03672188	2	Recruiting	Vir Biotechnology, Inc.
	DCR-HBVS	NCT03772249	1	Recruiting	Dicerna Pharmaceuticals
	JNJ-3989 (formely ARO-HBV)	NCT03365947	1	Recruiting	Arrowhead Pharmaceuticals
		NCT04208386	1	Recruiting	Janssen Sciences
	ARB-1467	NCT02631096	2	Completed	Arbutus Biopharma Co
**Antisense oligonucleotides**	GSK3389404	NCT03020745	2	Completed	GlaxoSmithKline
	RO7062931 (also known as RG6004)	NCT03038113	2	Completed	Hoffmann-La Roche
	GSK 3,228,836 (IONIS-HBVRx)	NCT02981602	2	Completed	GlaxoSmithKline
**Capsid inhibitors**	GLS4 (Morphothiadin mesilate/ritonavir)	NCT03638076	2	Recruiting	Sunshine Lake Pharma
	JNJ 56136379	NCT03361956	2	Active, not recruiting	Janssen Sciences
	JNJ 56,136,379 + JNJ 73763989	NCT04129554	2	Recruiting	Janssen Sciences
	JNJ 56,136,379 + JNJ 73763989	NCT03982186	2	Recruiting	Janssen Sciences
	ABI-H0731	NCT03577171	2	Completed	Assembly Biosciences
		NCT03576066	2	Completed	Assembly Biosciences
		NCT03780543	2	Active, not recruiting	Assembly Biosciences
	ABI-H2158	NCT03714152	1	Recruiting	Assembly Biosciences
		NCT04083716	1	Completed	Assembly Biosciences
	QL-007	NCT04157699	2	Recruiting	Qilu Pharmaceutical
		NCT04157257	2	Recruiting	Qilu Pharmaceutical
	RO7049389 (also known as RG7907)	NCT02952924	1	Recruiting	Hoffmann-La Roche
	EDP-514	NCT04008004	1	Recruiting	Enanta Pharmaceuticals
**Capsid inhibitor + TLR7**	RO7049389 + RO7020531 (also known as RG7854)	NCT04225715	2	Not yet recruiting	Hoffmann-La Roche
**HBsAg release inhibitors**	REP 2139	NCT02876419	2	Active, not recruiting	Replicor Inc.
	REP 2139-Mg + REP 2165-Mg	NCT02565719	2	Completed	Replicor Inc.
	REP 2139-Ca	NCT02726789	2	Completed	Replicor Inc.
	REP 2139-Ca	NCT02233075	2	Completed	Replicor Inc.

Note: No preclinical data are included. Only clinical trials completed within the last 2 years are included. Data are drawn from ClinicalTrials.gov.

**Table 2:** Novel immunotherapies in clinical trials in chronic HBV infection.

Drug Family	Drug Name	Trial Number	Phase	Status	Sponsor
Therapeutic vaccines	JNJ-64300535	NCT03463369	1	Recruiting	Janssen Sciences
	FP-02.2	NCT02496897	1	Completed	Altimmune, Inc.
	DV-601	NCT01023230	1	Completed	Dynavax Technologies Co
	INO-1800 with orwWithout INO-9112	NCT02431312	1	Completed	Inovio Pharmaceuticals
	Multiple molecules	NCT03866187	1	Recruiting	GlaxoSmithKline
	HBV0003	NCT03038802	1	Not yet recruiting	Vaxine Pty Ltd.
	TG1050	NCT04168333	1	Completed	Tasly Tianjin Biopharmaceutical
		NCT02428400	1	Completed	Transgene
	GS4774	NCT01943799	2	Completed	Gilead Sciences
		NCT02174276	2	Completed	Gilead Sciences
	T101 (therapeutic HB Adenovirus)	NCT04168333	1	Completed	Tasly Tianjin Biopharmaceutical
	ABX203	NCT02249988	3	Completed	Abivax S.A.
Toll-like receptors agonists	GS9688 (Selgantolimod)	NCT03491553	2	Active	Gilead Sciences
		NCT03615066	2	Active	Gilead Sciences
	GS-9620 (Vesatolimod)	NCT02579382	2	Completed	Gilead Sciences
		NCT02166047	2	Completed	Gilead Sciences
	RO7020531 (also nknown as RG7854)	NCT02956850	1	Recruiting	Hoffmann-La Roche
	TQ-A3334	NCT04180150	2	Recruiting	Chia Tai Tianqing
	RO6864018 (also known as RG7795)	NCT02391805	2	Completed	Hoffmann-La Roche
Apoptosis inducer	APG-1387	NCT03585322	1	Active, not recruiting	Ascentage Pharma
RIG-I agonists	Inarigivir Soproxil (GS-9992)	NCT03434353	2	Active, not recruiting	Gilead Sciences
		NCT04059198	2	Active, not recruiting	Gilead Sciences
	SB-9200	NCT02751996	2	Completed	Spring Bank Pharmaceuticals
Ciclophilin inhibitor	CRV-31	NCT03596697	1	Active, not recruiting	ContraVir Pharmaceuticals
Monoclonal anti-HBsAg antibody	GC1102	NCT03801798	2	Active, not recruiting	Green Cross Corporation
Unknown mechanism of action	RO7239958 (also known as RG6217)	NCT03762681	1	Recruiting	Hoffmann-La Roche
Anti-programmed cell death-1 (PD-1) humanized monoclonal antibody	HLX10	NCT04133259	2	Not yet recruiting	Henlix, Inc.
	Cemiplimab	NCT04046107	2	Recruiting	National Institute of Allergy Inf Dis

Note: No preclinical data are included. Only clinical trials completed within the last 2 years are included. Data are drawn from ClinicalTrials.gov.
